# Ghrelin Protects against Dexamethasone-Induced INS-1 Cell Apoptosis via ERK and p38MAPK Signaling

**DOI:** 10.1155/2016/4513051

**Published:** 2016-04-12

**Authors:** Chengshuo Zhang, Le Li, Bochao Zhao, Ao Jiao, Xin Li, Ning Sun, Jialin Zhang

**Affiliations:** ^1^Hepatobiliary Surgery Department and Unit of Organ Transplantation, First Hospital of China Medical University, Shenyang 110001, China; ^2^Hepatobiliary Surgery Department, Chifeng Municipal Hospital, Chifeng 024000, China; ^3^Department of General Surgery, Fourth Hospital of China Medical University, Shenyang 110032, China

## Abstract

Glucocorticoid excess induces apoptosis of islet cells, which may result in diabetes. In this study, we investigated the protective effect of ghrelin on dexamethasone-induced INS-1 cell apoptosis. Our data showed that ghrelin (0.1 *μ*M) inhibited dexamethasone-induced (0.1 *μ*M) apoptosis of INS-1 cells and facilitated cell proliferation. Moreover, ghrelin upregulated Bcl-2 expression, downregulated Bax expression, and decreased caspase-3 activity. The protective effect of ghrelin against dexamethasone-induced INS-1 cell apoptosis was mediated via growth hormone secretagogue receptor 1a. Further studies revealed that ghrelin increased ERK activation and decreased p38MAPK expression after dexamethasone treatment. Ghrelin-mediated protection of dexamethasone-induced apoptosis of INS-1 cells was attenuated using the ERK inhibitor U0126 (10 *μ*M), and cell viability increased using the p38MAPK inhibitor SB203580 (10 *μ*M). In conclusion, ghrelin could protect against dexamethasone-induced INS-1 cell apoptosis, at least partially via GHS-R1a and the signaling pathway of ERK and p38MAPK.

## 1. Introduction

Glucocorticoids are widely used in the clinic for the treatment of autoimmune and inflammatory diseases. However, long-term and high-dose glucocorticoid treatment has adverse effects and can induce glucose intolerance and steroid diabetes [1]. In general, glucocorticoid-induced hyperglycemia is partially caused by an increase in hepatic gluconeogenesis and resistance to insulin [2]. In addition, glucocorticoids are known to inhibit insulin secretion, which involves activating *α*_2_-adrenocortical signaling [3], increasing voltage gated K channels activity [4], and reducing glucose metabolism [5]. Moreover, considerable evidence has demonstrated that dexamethasone causes apoptosis of islet cells [2, 6], which is implicated in the activation of glucocorticoid receptors and calcineurin, and causes an imbalance in the anti- and proapoptotic proteins Bcl-2 and BAD, respectively. Therefore, exploration of efficient therapeutic agents inhibiting islet cell apoptosis following glucocorticoid administration has become a field of interest.

Ghrelin, a 28-amino acid polypeptide, was initially isolated from the human and rat stomach as an endogenous ligand of growth hormone secretagogue receptor type 1a (GHS-R1a) [7]. A number of studies have revealed that administration of exogenous ghrelin produces pleiotropic actions, including the capacity to decrease energy expenditure, increase food intake and adiposity, and facilitate gastric motility and glucose metabolism [7–11]. Moreover, some studies have reported that ghrelin raises blood glucose, which is attributed to the capacity of ghrelin to trigger growth hormone (GH) feeding, decrease insulin sensitivity, and suppress glucose-stimulated insulin secretion in *β* cells and directly stimulate the release of glucagon from *α* cells [12, 13]. However, an antiapoptotic effect of ghrelin has been observed in several animal and cell models. Ghrelin has been shown to exert a protective effect in both human islet and *β* cells, inhibit cell apoptosis, and modulate the expression of genes that are essential in pancreatic islet cell biology [14, 15]. In addition, ghrelin can directly stimulate *β*-cell proliferation* in vivo* without exerting its orexigenic or GH-stimulating properties in a streptozotocin- (STZ-) induced diabetic rat model [16]. However, whether ghrelin exerts a protective effect on dexamethasone-induced islet cell apoptosis remains unclear.

Thus, we aimed to explore whether ghrelin is able to protect INS-1 cells from dexamethasone-induced cell apoptosis and determine its mechanism of action. We show that ghrelin facilitates cell proliferation and significantly inhibits dexamethasone-induced apoptosis of INS-1 cells. In addition, we reveal that the antiapoptotic effect of ghrelin is mediated partially by activating the ERK signaling pathway and inhibiting the p38MAPK pathway, which was mediated through the GHS-R1a.

## 2. Materials and Methods

### 2.1. Cell Culture

INS-1 cells (Bioleaf Biotech Co., Ltd., Shanghai, China), derived from a rat insulinoma, were cultured in RPMI 1640 (GIBCO, California, USA) supplemented with 10% (v/v) fetal calf serum (GIBCO), 10 *μ*M *β*-mercaptoethanol, 10 mM HEPES, 2 mM L-glutamine, 1 mM Na pyruvate, 100 U/mL penicillin, and 100 *μ*g/mL streptomycin, as described previously [17]. The cells were maintained at 37°C in a humidified atmosphere containing 95% air and 5% CO_2_.

### 2.2. Drug Treatment

Dexamethasone (Sigma, Shanghai, China), U0126 (Selleckchem, Houston, TX, USA), and SB203580 (Cayman, Ann Arbor, MI, USA) were dissolved in dimethyl sulfoxide (DMSO) and added to complete medium containing a final concentration of 0.1% (v/v) DMSO. Acylated ghrelin (Anaspec, Fremont, CA, USA) was dissolved in phosphate buffer solution (PBS) and cells were pretreated for 1 h before exposure to dexamethasone. The antagonist of ghrelin receptor, [D-Lys^3^]-GH-releasing peptide- (GHRP-) 6 (100 *μ*M, Sigma, Shanghai, China), was dissolved in PBS and treated for 1 h before ghrelin disposal. For control, PBS without ghrelin or [D-Lys^3^]-GHRP-6 was used. For some experiments, cells were pretreated with the ERK inhibitor U0126 (10 *μ*M) or the p38MAPK inhibitor SB203580 (10 *μ*M) for 15 min before dexamethasone treatment. Control cells were treated with media containing 0.1% (v/v) DMSO without U0126 or SB203580.

### 2.3. Cell Viability Assay

Cell viability was measured using the CellTiter 96 Aqueous One Solution cell proliferation assay to monitor the living cells (Promega, Madison, WI, USA) according to the manufacturer's instructions. Briefly, cells were seeded in 96-well plates at a concentration of 1 × 10^4^ cells/well, allowed to adhere overnight, and subsequently exposed to different complete media (100 *μ*L) with or without dexamethasone or the other compounds at the indicated concentrations for 48 h. After 20 *μ*L one solution reagent was added, the absorbance value at 490 nm was measured using an ELISA reader (BioTek, VT, USA). The viability ratio was calculated according to the following formula: viability ratio = [(absorbance of experimental group − absorbance of blank group)/(absorbance of control group − absorbance of blank group)] × 100%.

### 2.4. 5-Ethynyl-2′-deoxyuridine (EdU) Incorporation Assay

Proliferating cells were stained with EdU using the Cell-Light EdU DNA Cell Proliferation Kit (RIBOBio Co., Guangzhou, China). Briefly, 5 × 10^3^ cells/well were seeded in 96-well plates and maintained as indicated above. After 48 h, 50 *μ*mol/L of EdU was added to cells for 4 h at 37°C. After fixation with 4% (w/v) paraformaldehyde for 30 min, the cells were treated with 0.5% (v/v) Triton X-100 for 20 min and rinsed with PBS three times. Thereafter, the cells were exposed to 100 *μ*L of 1x Apollo® reaction cocktail for 30 min and incubated with 5 *μ*g/mL of Hoechst 33342 to stain the cell nuclei for 30 min. EdU-labeled cells and Hoechst 33342-stained cells were counted in 10 random fields of view using a fluorescent microscope (Olympus IX71, Tokyo, Japan). The percentage of EdU-positive cells was calculated as the number of Hoechst-positive cells/the number of Hoechst-positive cells.

### 2.5. Flow Cytometry Analysis

Cell apoptosis was determined using the annexin V-FITC apoptosis detection kit (Bio-science, Beijing, China). Briefly, 2 × 10^5^ cells/well were seeded in 6-well plates. After 48 h, all adherent cells were collected with 0.25% (w/v) trypsin without EDTA, including floating cells in the medium. Annexin V-FITC and propidium iodide (PI) were used to label cells according to the manufacturer's instructions. The double-stained cells were subsequently analyzed by a FACSCanto flow cytometer (Becton-Dickinson, Mountain View, CA, USA). The cells stained with both PI and annexin V were considered to be late apoptotic, and the cells stained only with annexin V were considered to be early apoptotic. At least 10,000 cells were counted each time.

### 2.6. Caspase-3 Assay

Caspase-3 activation was determined using the Caspase-3 Activity Assay Kit (Beyotime, Haimen, China) according to the manufacturer's instructions. Briefly, cells were exposed to test substances for 48 h. Culture medium was removed, and cells were resuspended in lysis buffer after washing with ice-cold PBS and then incubated on ice for 15 min. After centrifugation at 14,000 ×g for 15 min, the supernatant was transferred to a fresh tube. Caspase-3 activity was determined using a colorimetric activity assay, which is based on spectrophotometric detection of p-nitroaniline (pNA) after catalysis from the labeled substrate Ac-DEVD-pNA. We quantified free pNA at 405 nm using an enzyme-linked immunosorbent assay reader (BioTek).

### 2.7. Western Blot Analysis

Cells treated as indicated above were washed twice with ice-cold PBS and lysed in ice-cold western and IP cell protein lysis buffer (P0013, Beyotime) supplemented with 1% (v/v) protease inhibitor cocktail and phenylmethanesulfonyl fluoride. The lysates were centrifuged at 12,000 ×g for 10 min at 4°C. Protein concentrations were determined using a BCA Protein Assay Kit (Beyotime) and samples were then denatured by boiling. Total protein (25 *μ*g/lane) was resolved using sodium dodecyl sulfate-polyacrylamide gel electrophoresis (SDS-PAGE) and transferred onto a polyvinylidene fluoride (PVDF) membrane using a wet transfer system (Bio-Rad, USA) at 70 V and 4°C. For immunoblotting, the PVDF membrane was incubated with Tris-buffered saline containing 0.1% (v/v) Tween-20 (TBS-T) and 5% (w/v) nonfat milk for 1 h, followed by incubation with the primary antibody overnight at 4°C. Horseradish peroxidase- (HRP-) conjugated IgG was used as the secondary antibody and membranes were incubated for 2 h. Afterwards, reactive protein was detected using an enhanced chemiluminescence (ECL) kit (Beyotime). The results were recorded using the MicroChemi Bio-Imaging System (DNR Bio-Imaging Systems Ltd., Jerusalem, Israel) and Quantity One version 4.5.0 software (Bio-Rad, Hercules, CA, USA). The primary antibodies used for this study were as follows: rabbit anti-cleaved-caspase-3 polyclonal antibody (1 : 500; Cat number 25546-1-AP; Proteintech Group, Inc., Chicago, IL, USA), rabbit anti-Bcl-2 polyclonal antibody (1 : 500; Cat number 12789-1-AP; Proteintech Group), mouse anti-Bax polyclonal antibody (1 : 500; Cat number 60267-1-Ig; Proteintech Group), goat anti-GHS-R1a polyclonal antibody (1 : 500; Cat number 10359; Santa Cruz Biotechnology, Inc., Santa Cruz, CA, USA), rabbit anti-ERK polyclonal antibody (1 : 500; Cat number 94; Santa Cruz Biotechnology), rabbit anti-p38MAPK polyclonal antibody (1 : 500; Cat number 535; Santa Cruz Biotechnology), rabbit anti-JNK polyclonal antibody (1 : 500; Cat number 571; Santa Cruz Biotechnology), mouse anti-p-ERK monoclonal antibody (1 : 500; Cat number 7383; Santa Cruz Biotechnology), mouse anti-p-p38MAPK monoclonal antibody (1 : 500; Cat number 7973; Santa Cruz Biotechnology), mouse anti-pJNK monoclonal antibody (1 : 500; Cat number 6254; Santa Cruz Biotechnology), and mouse anti-tubulin monoclonal antibody (1 : 2000; Cat number 0098; Cwbiotech, Beijing, China). The secondary antibodies included goat anti-rabbit IgG serum (1 : 40000; Zhongshan Golden Bridge, Beijing, China) and goat anti-mouse IgG serum (1 : 40000; Zhongshan Golden Bridge).

### 2.8. Statistical Analysis

All experiments were performed three times. Data were presented as means ± standard deviations (SD). The differences were analyzed using one-way ANOVA followed by the Student-Newman-Keuls test and all statistical analyses were performed using GraphPad Prism 5 (GraphPad Software, Inc., USA). Statistical differences were presented at probability levels of *p* < 0.05, *p* < 0.01, and *p* < 0.001 (2-sided significance testing).

## 3. Results

### 3.1. Ghrelin Inhibits Dexamethasone-Induced Cytotoxicity of INS-1 Cells

**Figure 1:**
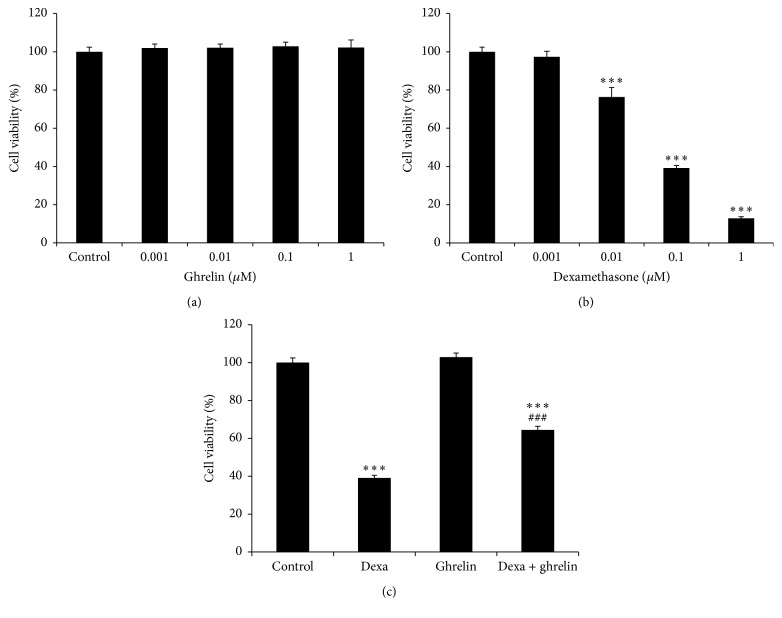
Effects of ghrelin and dexamethasone on INS-1 cell viability. (a, b) INS-1 cells were seeded at a density of 1 × 10^4^ cells/well in 96-well plates and treated with 0.001–1 *μ*M ghrelin or dexamethasone for 48 h. Cell viability was assessed using the MTS assay. (c) Ghrelin protects INS-1 cells from dexamethasone-induced cell apoptosis. INS-1 cells were incubated in complete medium (control), 0.1 *μ*M dexamethasone, 0.1 *μ*M ghrelin, and 0.1 *μ*M dexamethasone + 0.1 *μ*M ghrelin for 48 h. The Promega cell proliferation assay was used to determine the viability of INS-1 cells. The data are presented as the mean ± SD. ^*∗∗∗*^ *p* < 0.001 versus control group; ^###^ *p* < 0.001 versus dexamethasone group.

To determine the working concentration of ghrelin, INS-1 cells were treated with 0.001, 0.01, 0.1, and 1 *μ*M ghrelin. None of the concentrations tested had any marked effect on cell viability (Figure 1(a)). In addition, we performed a series of dose-dependent assays to assess the cytotoxicity of dexamethasone on INS-1 cells. Cells were exposed to 0.001, 0.01, 0.1, and 1 *μ*M dexamethasone and cell viability was determined at 48 h. As shown in Figure 1(b), dexamethasone treatment gradually induced a dose-dependent reduction in cell viability with an IC_50_ value of 0.092 *μ*M. Therefore, we chose a concentration of 0.1 *μ*M for both dexamethasone and ghrelin for subsequent experiments. Next, we examined the effect of ghrelin on dexamethasone-induced cytotoxicity in INS-1 cultures. As shown in Figure 1(c), ghrelin significantly attenuated dexamethasone-induced cell death of INS-1 cells compared with dexamethasone-only-treated cells.

### 3.2. Ghrelin Stimulates Proliferation in Dexamethasone-Treated INS-1 Cells

**Figure 2:**
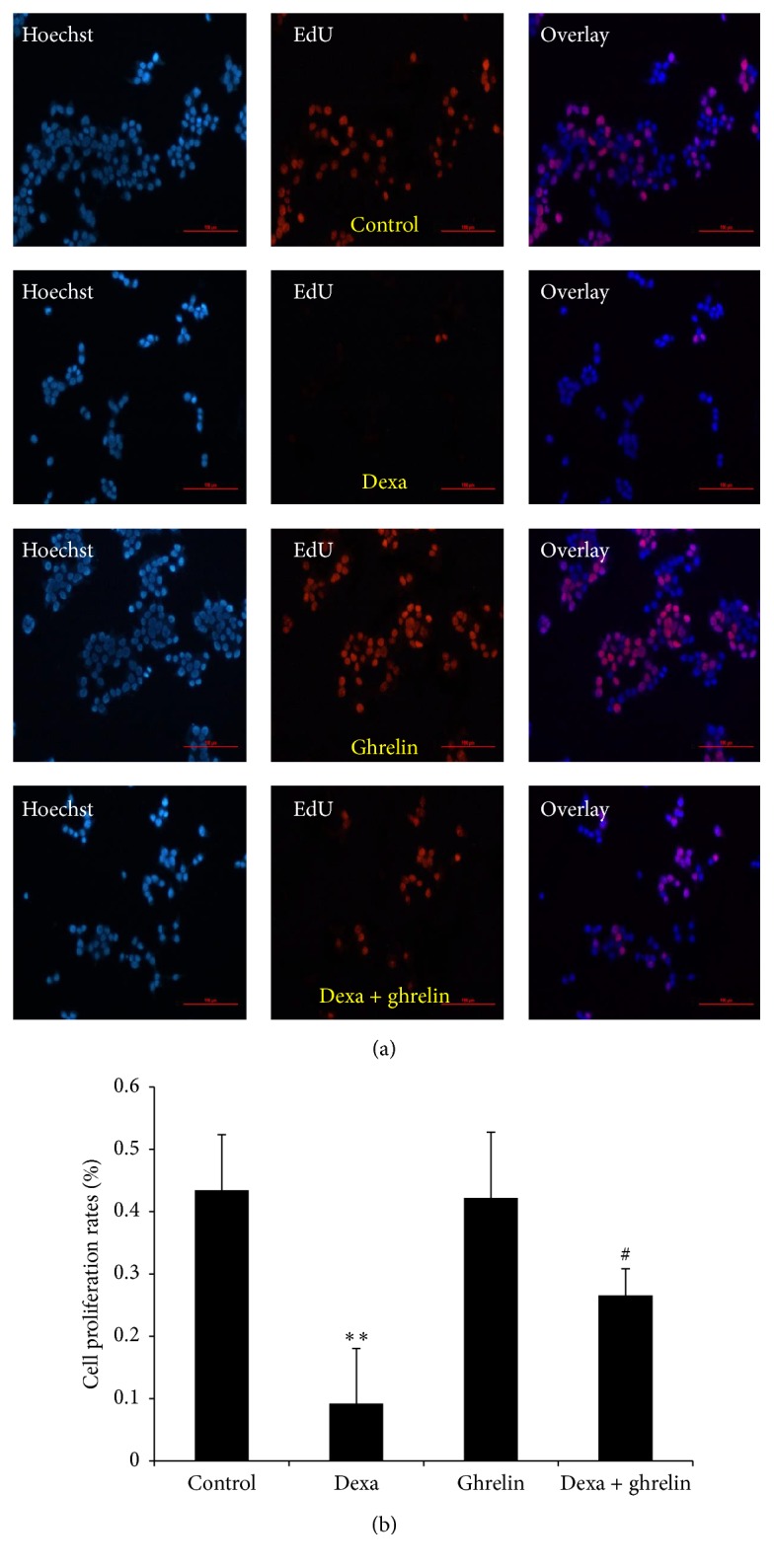
Effects of ghrelin and dexamethasone on DNA synthesis in INS-1 cells. (a) INS-1 cells were cultured in complete medium (control), 0.1 *μ*M dexamethasone, 0.1 *μ*M ghrelin, and 0.1 *μ*M dexamethasone + 0.1 *μ*M ghrelin for 48 h. EdU-labeled INS-1 cells were assessed using fluorescent microscopy. All cell nuclei were Hoechst 33342-positive (blue) and all replicating cells were EdU-positive (red). (b) The graph represents the percentage of EdU-labeled proliferative INS-1 cells. Data are presented as the mean ± SD. ^*∗∗*^ *p* < 0.01 versus control group; ^#^ *p* < 0.05 versus dexamethasone group.

EdU, a thymidine analog in which a terminal alkyne group substitutes the methyl group in the 5th position, is incorporated into cellular DNA during DNA replication in proliferating cells [18]. EdU labeling was performed to determine the effect of ghrelin on cell proliferation in dexamethasone-treated INS-1 cells. As shown in Figures 2(a) and 2(b), ghrelin significantly restored DNA synthesis after dexamethasone treatment compared with dexamethasone-only-treated cells, and more cells showed red fluorescence, indicating EdU labeling.

### 3.3. Ghrelin Inhibits Dexamethasone-Induced Mitochondrial-Dependent Apoptosis of INS-1 Cells

**Figure 3:**
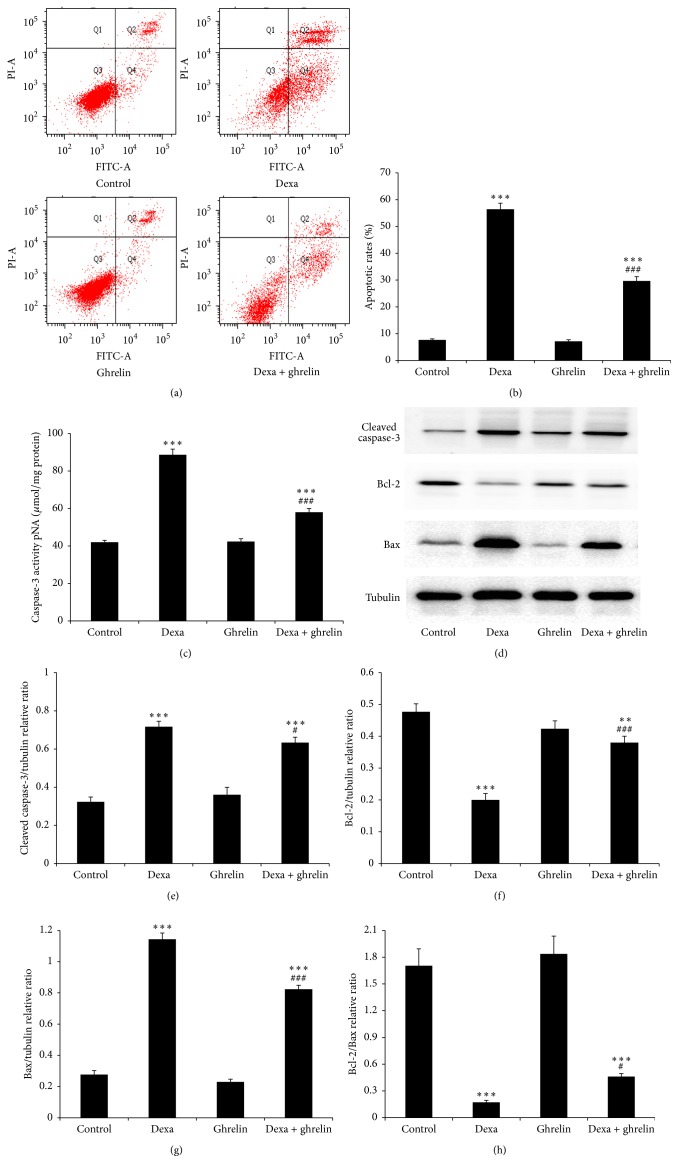
Ghrelin inhibits dexamethasone-induced mitochondrial-dependent apoptosis of INS-1 cells. (a) Extent of apoptosis following treatment with dexamethasone and ghrelin. INS-1 cells were cultured in complete medium (control), 0.1 *μ*M dexamethasone, 0.1 *μ*M ghrelin, and 0.1 *μ*M dexamethasone + 0.1 *μ*M ghrelin for 48 h. INS-1 cells were then stained with PI and annexin V-FITC and sorted by fluorescence flow cytometry. (b) The graph presents the percentage of apoptotic INS-1 cells. (c) Ghrelin suppressed the activation of caspase-3 in dexamethasone-treated INS-1 cells. Caspase-3 activity was measured using the caspase-3 colorimetric activity assay kit. (d) The expression of cleaved caspase-3, Bcl-2, and Bax in treated INS-1 cells was detected using western blot analysis. (e–h) Graphs indicate the ratio between the bands of interest. Data are presented as the mean ± SD. ^*∗∗∗*^ *p* < 0.001, ^*∗∗*^ *p* < 0.01 versus control group; ^###^ *p* < 0.001, ^#^ *p* < 0.05 versus dexamethasone group.

We employed flow cytometry to detect INS-1 apoptotic cell death after dexamethasone and ghrelin treatment. As shown in Figures 3(a) and 3(b), the percentage of apoptotic INS-1 cells was significantly increased after dexamethasone treatment, and ghrelin significantly reduced the number of dexamethasone-induced apoptotic cells (dexamethasone 56.4 ± 2.3% versus dexamethasone + ghrelin, 29.7 ± 1.6%).

Moreover, a significant increase in caspase-3 activity was also detected in INS-1 cells treated with dexamethasone compared with the control (dexamethasone 88.7 ± 3.1 *μ*mol/mg protein versus control 42.0 ± 1.0 *μ*mol/mg protein). When INS-1 cells were cocultured with dexamethasone and ghrelin for 48 h, caspase-3 activity decreased to 58.0 ± 2.0 *μ*mol/mg protein (Figure 3(c)).

Caspase-3 activity, which positively correlates with cell apoptosis, is a pivotal biomarker of apoptosis. The imbalance in Bcl-2 and Bax expression is closely related to mitochondrial apoptosis. As shown in Figures 3(d)–3(h), the level of cleaved caspase-3 (17 kDa) increased after dexamethasone treatment, while cotreatment with ghrelin significantly inhibited activation. Furthermore, ghrelin significantly prevented the dexamethasone-mediated downregulation of Bcl-2 (27 kDa) and upregulation of Bax (24 kDa), which resulted in an increase in the Bcl-2/Bax ratio. All these results indicate that ghrelin reduces dexamethasone-induced INS-1 cell apoptosis via mitochondrial-dependent processes.

### 3.4. The Protective Effect of Ghrelin Is Mediated through Ghrelin Receptor, GHS-R1a, in INS-1 Cells

**Figure 4:**
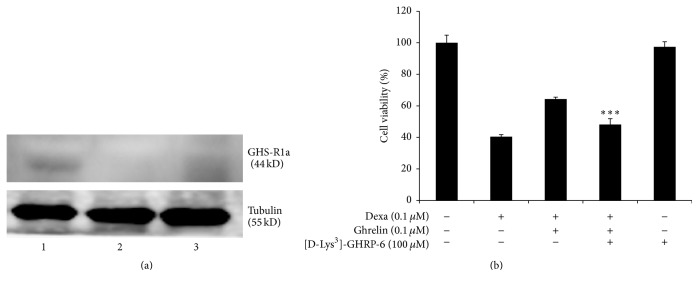
The protective effect of ghrelin on apoptosis of INS-1 after dexamethasone treatment is mediated via the ghrelin receptor, GHS-R1a. (a) The expression of ghrelin receptor, GHS-R1a, was determined by western blot in rat INS-1 cells. Lane 1: rat hypothalamus, lane 2: rat BRL3A cells, and lane 3: rat INS-1 cells. (b) INS-1 cells were pretreated with control or ghrelin (0.1 *μ*M) for 1 h before dexamethasone treatment. The cells were also preincubated with PBS control or ghrelin receptor antagonist, [D-Lys^3^]-GHRP-6 (100 *μ*M), 1 h before ghrelin treatment. Cell viability was measured at 48 h after dexamethasone treatment by MTS assay. Cell survival was expressed as a percentage relative to that in the vehicle control (100%). The data are presented as the mean ± SD. ^*∗∗∗*^ *p* < 0.01 versus dexa + ghrelin group.

It has been known that the active effect of ghrelin is mainly mediated by ghrelin receptor, GHS-R1a, a G protein-coupled receptor located mainly in the hypothalamus [7]. We first performed western blot analysis to ascertain the expression of GHS-R1a in INS-1 cells. Next, to test the hypothesis that the antiapoptotic effect of ghrelin on INS-1 is mediated via the GHS-R1a, INS-1 cells were pretreated with the receptor antagonist [D-Lys^3^]-GHRP-6 before ghrelin treatment. As shown in Figure 4(a), ghrelin receptor, GHS-R1a, was expressed in INS-1 cells in comparison to the GHS-R1a extracted from rat hypothalamus. Exposure of cells to [D-Lys^3^]-GHRP-6 (100 *μ*M) abated the protective effect of ghrelin on INS-1 cells after dexamethasone treatment in the MTS assay (dexamethasone, 40.4 ± 1.3%; dexamethasone + ghrelin, 64.3 ± 1.3%; dexamethasone + ghrelin + [D-Lys^3^]-GHRP-6, 48.2 ± 3.7%). No cytotoxicity was detected when INS-1 was treated only with the ghrelin receptor antagonist. All these results indicate that the protective effect of ghrelin against dexamethasone-induced apoptosis of INS-1 cells is mediated via the ghrelin receptor, GHS-R1a.

### 3.5. The Protective Effect of Ghrelin Is Mediated by Increasing ERK Activation and Decreasing p38MAPK Expression after Dexamethasone Treatment

**Figure 5:**
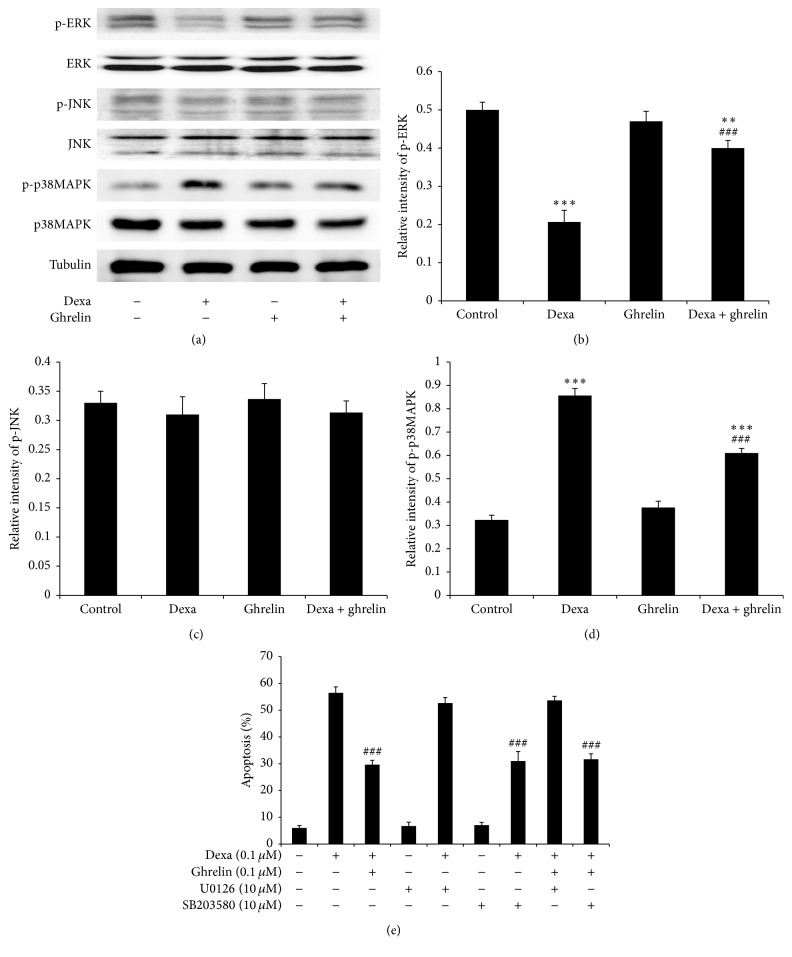
The effect of MAPK on dexamethasone-induced INS-1 cell apoptosis. (a) The effect of dexamethasone and ghrelin on MAPK expression in INS-1 cells. The expression of phospho-MAPK and MAPK in treated INS-1 cells was detected by western blot analysis. (b, c, d) Graphs indicate the quantitative analyses of pMAPK and MAPK. (e) The effect of ERK and p38MAPK inhibitors on INS-1 cell apoptosis after dexamethasone treatment. The ERK inhibitor U0126 (10 *μ*M) and p38MAPK inhibitor SB203580 (10 *μ*M) were added 15 min before dexamethasone (0.1 *μ*M) treatment. Cells were then stained with annexin V-FITC and PI and sorted by fluorescence flow cytometry after dexamethasone treatment for 48 h. Graphs represent the percentage of INS-1 cell apoptosis. Data are presented as the mean ± SD. ^*∗∗∗*^ *p* < 0.001, ^*∗∗*^ *p* < 0.01 versus control group; ^###^ *p* < 0.001 versus dexamethasone group.

The mitogen-activated protein kinase pathways (MAPK), including ERK, p38MAPK, and JNK, are known to be associated with cell death or survival [19]. Given that the protective effect of ghrelin has been linked to the MAPK signaling pathway* in vitro* and* in vivo* [16, 20–22], we investigated the effect of ghrelin on MAPK in INS-1 cells after dexamethasone treatment. Western blot analysis revealed that the level of p-ERK decreased and that the level of p-p38MAPK increased after dexamethasone treatment compared with the control (Figures 5(a), 5(b), and 5(d)). Moreover, the level of p-JNK did not significantly change following dexamethasone treatment (Figure 5(c)). The level of total ERK, p38MAPK, or JNK remained unchanged after dexamethasone treatment. Next, we determined the effect of ghrelin on ERK and p38MAPK expression after dexamethasone treatment. As shown in Figures 5(a)–5(d), ghrelin increased the level of p-ERK and decreased the level of p-p38MAPK after treatment with dexamethasone. Thus, these data suggest that the antiapoptotic effect of ghrelin on INS-1 cells after dexamethasone treatment may be mediated in part via the ERK and p38MAPK signaling pathway.

We assumed that ghrelin would promote INS-1 cell survival by increasing ERK activation and decreasing p38MAPK expression after dexamethasone treatment. INS-1 cells were pretreated for 15 min with the inhibitors of ERK (U0126) and p38MAPK (SB203580), followed by exposure to dexamethasone for 48 h. Annexin V/PI-double staining demonstrated that the number of apoptotic cells was significantly decreased by SB203580 + dexamethasone treatment when compared with dexamethasone-only-treated cells (dexamethasone + SB203580 31 ± 3.6% versus dexamethasone 56.4 ± 2.3%), whereas the protective effect of ghrelin was abolished by U0126 treatment (dexamethasone + ghrelin 29.7 ± 1.6% versus dexamethasone + ghrelin + U0126 53.7 ± 1.5% versus dexamethasone 56.4 ± 2.3%). However, there was no additional impact on cells treated with SB203580 after dexamethasone + ghrelin treatment (dexamethasone + ghrelin + SB203580 31.6 ± 2.1% versus dexamethasone + ghrelin 29.7 ± 1.6% versus dexamethasone 56.4 ± 2.3%). Moreover, INS-1 cell apoptosis was not affected when cells were treated with U0126 or SB203580 alone (Figure 5(e)). These results indicate that ERK activation is involved in cell survival and that p38MAPK activation is involved in INS-1 cell apoptosis after dexamethasone treatment. Moreover, our data indicate that the protective effect of ghrelin on INS-1 survival may be mediated partially by increasing ERK activation and decreasing p38MAPK expression after dexamethasone treatment.

## 4. Discussion

Although glucocorticoids have been reported to induce myocardium preservation and protect podocytes against apoptosis [23, 24], glucocorticoids are mainly known to lead to apoptotic cell death and reduce proliferation in a variety of cells, including INS-1 cells [2, 25]. Dexamethasone has been reported to increase reactive oxygen species production, decrease viability, and impair insulin secretion in pancreatic rat islets [26]. Ghrelin, a multifunctional polypeptide, has been known to promote cell proliferation and inhibit apoptosis via mitigating oxidative stress and preserving the activities of antioxidant enzymes, such as superoxide dismutase and catalase [27]. In view of this, we presumed that ghrelin may play a role in protecting islet INS-1 cells against dexamethasone-induced apoptosis. In this study, we demonstrated that ghrelin inhibited dexamethasone-induced INS-1 cell apoptosis and facilitated cell proliferation. Furthermore, we showed that ghrelin protected INS-1 cells from dexamethasone-induced apoptosis via restoring the ratio of Bcl-2/Bax and decreasing caspase-3 activation, and the protective effect of ghrelin was mediated through the ghrelin receptor, GHS-R1a. Finally, we proved that the antiapoptotic effect by ghrelin may be mediated partially by increasing ERK activation and decreasing p38MAPK expression after dexamethasone treatment.

In general, the ERK pathway plays an important role in cell proliferative and survival processes [28, 29]. Ghrelin has been shown to exert an antiapoptotic effect that is regulated by the ERK signaling pathway in oligodendrocytes, osteoblasts, and ovary cells [21, 22, 30]. Moreover, Bando et al. reported that the ERK pathway in human pancreatic islet microvascular endothelial cells is involved in the survival response after exposure to hyperglycemic conditions [16]. In this study, we demonstrated that ghrelin increased ERK activation in INS-1 cells after dexamethasone treatment and that inhibition of the ERK pathway by U0126 eliminates the protective effect of ghrelin on INS-1 cell survival. In summary, these results indicate that the ERK signaling pathway is involved in the INS-1 cell survival pathway, and ghrelin protects INS-1 cells by increasing ERK activation after dexamethasone treatment.

Some studies have shown that ghrelin facilitates cell survival by inhibiting p38MAPK expression in spinal cord injury and hydrogen peroxide toxicity [22, 31]. In the present study, we demonstrated that dexamethasone increased p38MAPK expression, which was inhibited by ghrelin treatment. Our data also revealed that dexamethasone-induced activation of p38MAPK was involved in the INS-1 cell apoptotic pathway. SB203580, an inhibitor of p38MAPK, increased cell survival after dexamethasone treatment. Because Zhao et al. reported that SB203580 inhibited the toxic effects of dexamethasone on acute lymphoblast leukemia cells [25], we hypothesized that the antiapoptotic effect of ghrelin on INS-1 cells may also involve inhibition of p38MAPK expression after dexamethasone treatment.

Our results reveal that the antiapoptotic effect of ghrelin may be mediated via the mitochondrial-dependent apoptotic pathway because ghrelin reversed the imbalance of Bcl-2 and Bax and inhibited caspase-3 activation in dexamethasone-induced INS-1 cells. Li et al. demonstrated that ghrelin inhibits cytochrome c release via protecting mitochondrial membrane depolarization and decreasing the Bax/Bcl-2 ratio during lipopolysaccharide-stimulated alveolar macrophages* in vitro* [20]. In addition, Xie et al. showed that ghrelin plays a neuroprotective role in a postresuscitation brain injury rat model of cardiac arrest by increasing the Bcl-2/Bax ratio and decreasing caspase-3 expression.

MAPK also has an effect on proteins in the mitochondrial apoptotic pathway [32]. The increase in ERK activation and/or decrease in p38MAPK expression has been reported to induce an increase in the ratio of Bcl-2/Bax [33, 34]. Moreover, dexamethasone has been demonstrated to induce INS-1 cell apoptosis via the mitochondrial pathway [2] and an imbalance in Bcl-2/BAD (Bax), which is a key point in the mitochondrial pathway. Therefore, we deduce that ghrelin inhibits apoptosis by increasing ERK activation and decreasing p38MAPK expression, which subsequently restores the ratio of Bcl-2/BAD (bax) and inhibits the activation of caspase-3.

The results of this study demonstrate that ghrelin may have beneficial effect on dexamethasone-induced INS-1 cells in the sense that it may decrease cell apoptosis and increase cell mass. However, the role of ghrelin in the regulation of insulin action and insulin secretion remains a dispute [35]. Although some reports found that administration of exogenous ghrelin suppresses insulin secretion and increases blood glucose level, inhibition of ghrelin alleviates glucose tolerance in mice by promoting insulin secretion [12, 13, 36, 37]. On the other side, ghrelin has also been shown to increase insulin secretion from the pancreas of both normal and diabetic rats and restore the insulin expression and secretion from STZ-induced newborn rats [16, 38, 39]. Therefore, we infer that ghrelin may express either stimulating or inhibiting insulin secretion depending upon the experimental condition, such as the ghrelin concentration. Further study is required to determine the effect of protective concentration of ghrelin on insulin secretion and glucose level.

In conclusion, our data reveal that ghrelin can inhibit dexamethasone-induced apoptosis of islet INS-1 cells, facilitate cell proliferation, and restore cell viability. Furthermore, the antiapoptotic effect of ghrelin is mediated via the ghrelin receptor and the protective effect of ghrelin is mediated partially by activating ERK signaling and inhibiting the p38MAPK pathway. Finally, our study suggests that ghrelin may be potentially useful as a therapeutic agent for preventing steroid diabetes mellitus when glucocorticoids are administered. However, the deleterious side of ghrelin's effect on *β* cells also should not be neglected.
